# Preoperative MRI-based radiomic nomogram for distinguishing solitary fibrous tumor from angiomatous meningioma: a multicenter study

**DOI:** 10.3389/fonc.2024.1399270

**Published:** 2024-09-18

**Authors:** Mengjie Li, Shengli Fu, Jingjing Du, Xiaoyu Han, Chongfeng Duan, Yande Ren, Yaqian Qiao, Yueshan Tang

**Affiliations:** ^1^ Department of Radiology, The Affiliated Hospital of Qingdao University, Qingdao, China; ^2^ Department of Radiology, Shizuishan First People's Hospital, Shizuishan, China; ^3^ Department of Radiology, Qilu Hospital, Shandong University, Jinan, China

**Keywords:** solitary fibrous tumor, angiomatous meningioma, radiomics, nomogram, machine learning

## Abstract

**Purpose:**

This study evaluates the efficacy of radiomics-based machine learning methodologies in differentiating solitary fibrous tumor (SFT) from angiomatous meningioma (AM).

**Materials and methods:**

A retrospective analysis was conducted on 171 pathologically confirmed cases (94 SFT and 77 AM) spanning from January 2009 to September 2020 across four institutions. The study comprised a training set (n=137) and a validation set (n=34). All patients underwent contrast-enhanced T1-weighted (CE-T1WI) and T2-weighted(T2WI) MRI scans, from which 1166 radiomics features were extracted. Subsequently, seventeen features were selected through minimum redundancy maximum relevance (mRMR) and the least absolute shrinkage and selection operator (LASSO). Multivariate logistic regression analysis was employed to assess the independence of these features as predictors. A clinical model, established via both univariate and multivariate logistic regression based on MRI morphological features, was integrated with the optimal radiomics model to formulate a radiomics nomogram. The performance of the models was assessed utilizing the area under the receiver operating characteristic curve (AUC), accuracy (ACC), sensitivity (SEN), specificity (SPE), positive predictive value (PPV), and negative predictive value (NPV).

**Results:**

The radiomics nomogram demonstrated exceptional discriminative performance in the validation set, achieving an AUC of 0.989. This outperformance was evident when compared to both the radiomics algorithm (AUC= 0.968) and the clinical model (AUC = 0.911) in the same validation sets. Notably, the radiomics nomogram exhibited impressive values for ACC, SEN, and SPE at 97.1%, 93.3%, and 100%, respectively, in the validation set.

**Conclusions:**

The machine learning-based radiomic nomogram proves to be highly effective in distinguishing between SFT and AM.

## 1 Introduction

Solitary fibrous tumor (SFT) represents a form of invasive soft tissue sarcoma (1). Previously grouped with hemangiopericytomas (HPC) under the term "solitary fibrous tumor/hemangiopericytoma" by the CNS WHO in 2016, the classification was revised in 2021, exclusively designating these lesions as SFT (2, 3). Meningioma, the most common adult intracranial tumor, is categorized into three WHO grades (3, 4). Angiomatous meningioma (AM), a grade 1 meningioma, poses diagnostic challenges owing to its histological resemblance to SFT (5, 6). Unlike AM, which generally has a favorable prognosis following surgical resection, SFT often manifests with extracranial metastasis and local recurrence (6–8). Accurate preoperative differentiation between these tumors is paramount for treatment planning.

Magnetic Resonance Imaging (MRI) is a primary tool for evaluating central nervous system malignancies (9). Contrast-Enhanced T1-Weighted Imaging (CE-T1WI) is instrumental in evaluating blood-brain barrier integrity and delineating tumor characteristics. T2-Weighted Imaging (T2WI) is valuable for superior soft tissue resolution in tumor detection (10). Preoperative imaging methods, including the intratumoral flow void sign and apparent diffusion coefficient (ADC) map, have been explored for differentiating SFT from AM (11, 12). However, these techniques are subjective and heavily reliant on radiologist expertise, underscoring the necessity for more objective and quantitative diagnostic approaches.

Radiomics, an emerging field in medical imaging, leverages data-characterization algorithms to extract quantitative features from radiological images, thereby augmenting prognostic monitoring and treatment strategies in oncology (13). Central to this domain, machine learning, particularly deep learning, plays a critical role in feature analysis, significantly contributing to advancements in medical imaging (14). By objectively assessing tumor heterogeneity, radiomics propels the development of precision oncology (15). Numerous studies have demonstrated that its application spans various cancer types, including stomach and esophageal cancers, and extends to intracranial tumors like gliomas and meningiomas (16, 17). In addition, radiomics has proven effective in differentiating between SFT and AM (5, 18, 19). According to Li et al. (19), the area under the receiver operating characteristic curve (AUC) of the CE-T1WI-based radiomics algorithm for distinguishing SFT and AM was 0.90, significantly higher than the AUCs of three neuroradiologists (AUC=0.69, 0.70, and 0.73). Nevertheless, MRI-based radiomics nomograms, integrating both conventional imaging features and radiomics for differentiating SFT and AM, remain underexplored.

Here, our research aims to ascertain the diagnostic performance of MRI-based radiomics nomogram in preoperative differentiation between SFT and AM, using data from four centers to enhance the precision of therapeutic decision-making.

## 2 Materials and methods

### 2.1 Study participants

The multicenter study was approved by the Institutional Review Board of our hospital, and written informed consent was waived on account of its retrospective nature. MRI data of SFT and AM were retrieved from picture archiving and communication systems via the radiology database. Patient recruitment occurred at four medical centers: the First Affiliated Hospital of Qingdao University (Medical Center A), Guangxi Medical University (Medical Center B), the Frist Affiliated Hospital of Zhengzhou University (Medical Center C), and Qilu Hospital of Shandong University (Medical Center D), over the time period extending from January 2009 to September 2020. The inclusion criteria were as follows: (1) pathological diagnosis of SFT or AM; (2) preoperative MRI examination performed without image artifacts; and (3) no prior treatment at the initial diagnosis. The exclusion criteria comprised: (1) artifacts on MRI images; (2) previous history of brain surgery or biopsy; and (3) previous history of intracranial diseases, such as subarachnoid hemorrhage or cerebral infarction.

**Figure 1:**
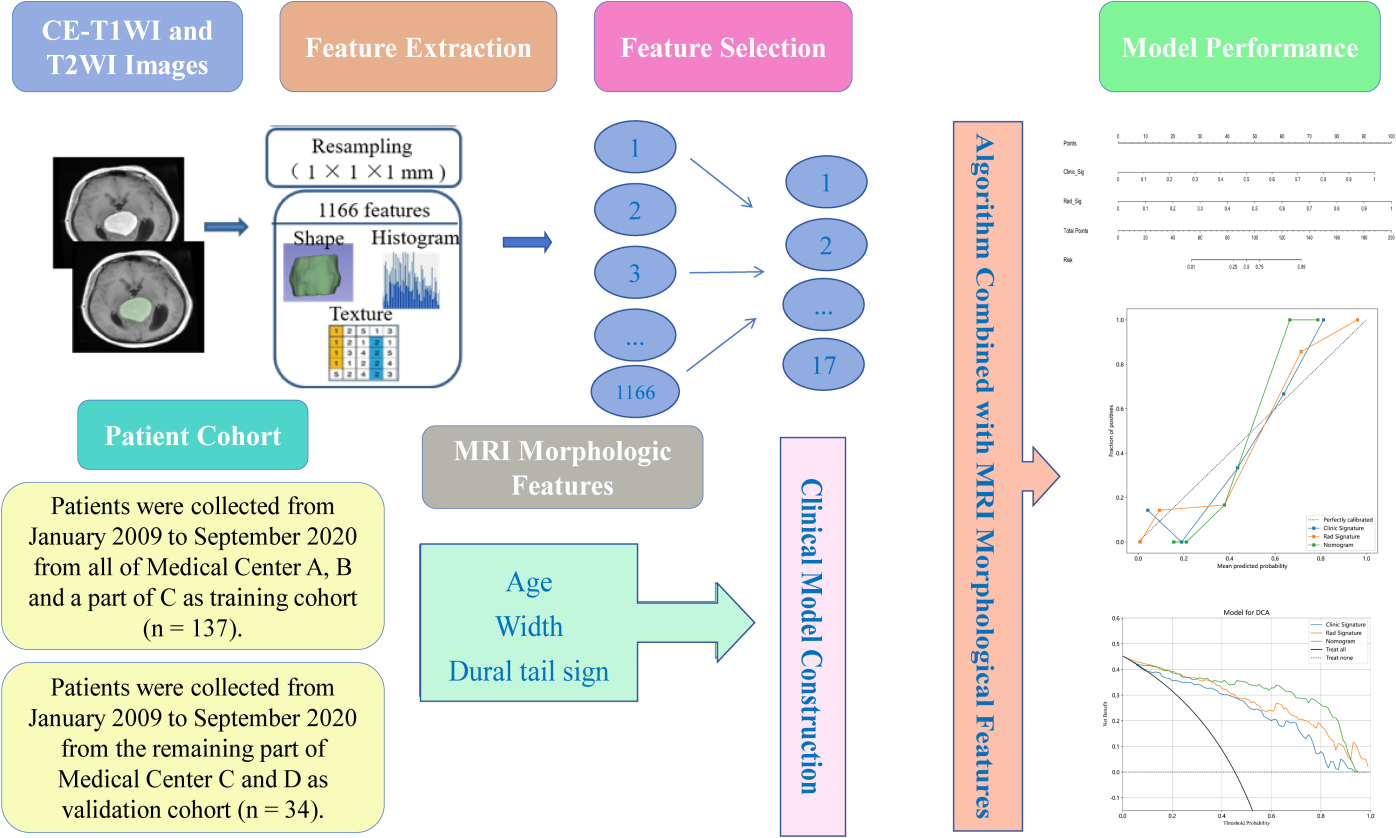
Workflow of the study.

Ultimately, the study comprised 94 patients with SFT and 77 with AM. The training cohort, selected from all of Medical Center A, B and a part of C, consisted of 75 SFT and 62 AM patients (n = 137), while the validation cohort from the remaining part of Medical Center C and D included 19 SFT and 15 AM cases (n = 34). The clinical characteristics of the 171 patients are shown in **Table 1**. The workflow of this study is illustrated in **Figure 1**.

**Table 1:** Patients' demographic information and morphological characteristics of SFT and AM in the training and validation sets.

characteristic	Training set (*n* = 137)			Validation set (*n* = 34)		
	SFT 75	AM 62	P value*	SFT 19	AM 15	p value*
Age(years) (mean ± SD)	46.77± 12.20	55.32± 10.70	<0.001	39.74± 16.20	52.87± 14.14	0.019
Size (mean ± SD)	46.35± 17.49	39.34± 12.06	0.008	60.23± 17.67	44.57± 12.10	0.006
Sex						
Male	37	37	0.300	12	5	0.167
Female	38	25		7	10	
Shape						
Defined	33	35		5	11	0.017
Ill-defined	42	27	0.201	14	4	
Dural tail sign						
Presence	22	53		6	14	0.001
Absence	53	9	<0.001	13	1	
Width						
Wide base	32	56		9	13	0.043
Narrow base	43	6	<0.001	10	2	
Cystic						
Presence	45	24		13	5	0.091
Absence	30	38	0.021	6	10	
Vessel flow voids						
Presence	46	38	1.000	14	13	0.615
Absence	29	24		5	2	
Edema						
Presence	53	43	1.000	15	13	0.894
Absence	22	19		4	2	

SFT, solitary fibrous tumor; AM, angiomatous meningioma; SD, standard deviation.

*Calculated from independent-sample t test for continuous variables and Fisher's exact or chi-square tests for categorical variables.

### 2.2 Image acquisition

The whole imaging data encompassed preoperative T2WI and CE-T1WI images, obtained using either a 3.0 T Siemens or a 3.0 T GE scanner. For CE-T1WI, images were acquired post-contrast injection through the cubital vein (gadopentetate dimeglumine, 0.1 mmol/kg), covering axial, coronal, and sagittal planes. The scanning parameters for each scanner were as follows: 3.0 T Siemens: relaxation time / echo time (TR/TE) 1800/8.5 ms; 3.0 T GE: TR/TE 2250/24 ms. The T2WI scanning parameters were: TR/TE, 700–6370/40–120 ms. The following imaging acquisition parameters were used for both Siemens and GE scanners: field of view (FOV) of 23 cm, slice thickness of 5 mm and slice gap of 1 mm.

### 2.3 MRI morphologic characteristics

Two experienced radiologists (7 and 25 years of neuroimaging experience, respectively) independently analyzed the images, blinded to the clinical data. Intraclass correlation coefficients (ICCs) were calculated to assess intraobserver reliability, with one radiologist repeatedly identifying signs, while interobserver reliability was determined by comparing analyses between different radiologists. For intraobserver reproducibility assessment, radiologist 1 conducted a second region of interest (ROI) delineation one week later. The morphologic signs evaluated included: (1) size (maximum diameter of mass); (2) shape (defined or ill-defined); (3) dural tail sign (presence or absence); (4) width (wide base or narrow base); (5) cystic area (presence or absence); (6) vessel flow voids (presence or absence); and (7) peritumoral edema (presence or absence).

### 2.4 ROI segmentation

The images were imported into 3D-slicer software (v.4.8.1, http://www.slicer.org/) in DICOM format. Utilizing axial images, two radiologists delineated the ROI along the tumor edge in a stepwise manner. After delineating the ROI on each slice around the tumor periphery, a three-dimensional (3D) ROI was constructed. The ROIs comprised cystic and hemorrhagic areas while avoiding the edema area, aorta, venous sinus and enhanced meninges (20). Any discrepancies were reconciled through discussion.

### 2.5 Image normalization and feature extraction

Due to the heterogeneity of the dataset, resulting from varying scanners and protocols, standardization processes such as resampling, noise reduction, and wavelet transform were applied to both CE-T1WI and T2WI images to minimize this impact. 3D Slicer was used for resampling to a voxel size of 1 × 1 × 1 mm and for performing Gaussian filtering with sigma values of 0.5, 1.0, and 1.5 (21). For further analysis, radiomics features with acceptable interobserver and intraobserver reproducibility (intraclass correlation coefficients [ICC] > 0.75) were chosen.

An internal MATLAB script (MATLAB R2017b, The MathWorks, Inc., Natick, MA, USA) was employed for the extraction of radiomics features in conjunction with 3D Slicer (22). A comprehensive set of 1,166 features, encompassing shape, first-order, gray level co-occurrence matrix (GLCM), gray level run length matrix (GLRLM), gray level size zone matrix (GLSZM), gray level dependence matrix (GLDM), and neighboring gray tone difference matrix (NGTDM) features, were extracted from CE-T1WI and T2WI images.

### 2.6 Feature selection

The Mann-Whitney U test and univariate logistic regression analysis were performed to examine whether these features were any significant differences between SFT and AM. To reduce redundancy in features, the least absolute shrinkage and selection operator (LASSO) and the minimum redundancy maximum relevance (mRMR) methods were applied. Only features exhibiting the highest predictive value and significant association with the differentiation of SFT and AM were retained. In this study, three machine-learning classifiers were employed: support vector machine (SVM), logistic regression (LR) and k-nearest neighbor (KNN).

### 2.7 Developing the radiomics model, adding the clinical model, and constructing the radiomics nomogram

Clinical morphology signs were selected through univariate logistic regression, and clinical features with P < 0.05 were incorporated into a multivariate logistic regression to develop a clinical model with backwards stepwise selection and Akaike’s information criterion as the stopping rule. Subsequently, radiomics features selected by mRMR and LASSO were combined with SVM, LR and KNN respectively to develop the radiomics models. Utilizing the best classifier and feature selection approach, a radiomics score (Rad-score) was determined. After that, combining the morphologic features identified through multivariable logistic regression analysis and the rad-score to construct a nomogram.

The performance of the models was evaluated by calculating the AUC, accuracy, sensitivity, specificity, negative predictive value (NPV), and positive predictive value (PPV). Calibration curves and decision curve analysis (DCA) were utilized for assessing the nomogram’s calibration ability and clinical utility, respectively. The dependability of models was evaluated at the net benefit level using DCA, with a higher standard net benefit indicating greater clinical applicability across various threshold probabilities (23).

### 2.8 Statistical analysis

All statistical analyses were executed using R statistical software (https://www.Rproject.org). The independent-sample t test assessed continuous variables (such as age), while categorical variables, like gender, were analyzed using Fisher's exact or chi-square tests. A value of P < 0.05 was considered statistically significant.

## 3 Results

### 3.1 Clinical characteristics screening and model development

**Figure 2:**
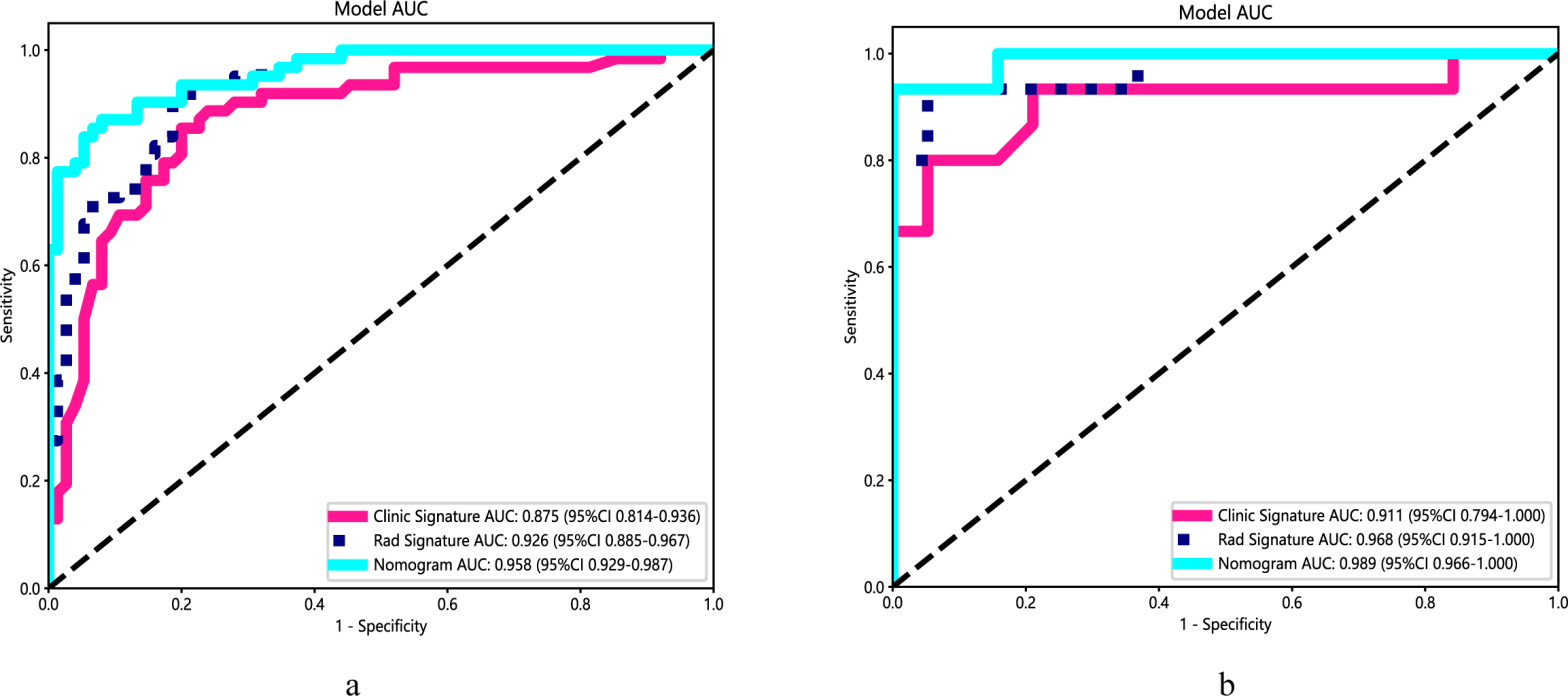
Performance of the clinical model, radiomics algorithm and the radiomics nomogram. **(A)** Training set. **(B)** Validation set.

**Table 1** presents the clinical characteristics of the training set and validation set. The assessment of intraobserver and interobserver reliability yielded ICCs for MRI morphological features that consistently exceeded 0.75. Univariate and multivariate logistic regression analysis results are presented in **Table 2**. Notably, multivariate logistic regression analysis identified age, width, and dural tail sign as independent risk factors for discriminating between SFT and AM. A clinical model incorporating these variables was developed, demonstrating AUCs of 0.875 (95% confidence interval [CI], 0.814-0.936) in the training set and 0.911 (95% CI, 0.794-1.000) in the validation set. A list of results is displayed in **Table 3** and **Figure 2**.

**Table 2:** Results of univariate and multivariate logistic regression analysis in SFT and AM.

Variable	Univariate Analysis			Multivariate Analysis		
	OR	(95% CI)	*P*-value	OR	(95% CI)	*P*-value
Shape	0.826	[0.729;0.935]	0.012	0.941	[0.850;1.041]	0.318
Dural tail sign	1.775	[1.598;1.970]	<0.001	1.428	[1.267;1.610]	<0.001
Width	1.642	[1.462;1.844]	<0.001	1.312	[1.163;1.481]	<0.001
Cystic	0.788	[0.697;0.891]	0.002	0.890	[0.797;0.994]	0.083
Age	1.013	[1.009;1.018]	<0.001	1.008	[1.004;1.012]	<0.001
Size	0.992	[0.988;0.996]	<0.001	0.997	[0.993;1.000]	0.112

OR, odds ratio; CI, confidence interval.

**Table 3:** Results of Clinical Model, Radiomics Algorithm and the Radiomics Nomogram Predictive Performance.

Group		AUC	95% CI	ACC	SEN	SPE	PPV	NPV
**Training set**	Clinical	0.875	0.814 0.936	0.825	0.855	0.800	0.779	0.870
	Algorithm	0.926	0.885-0.967	0.854	0.935	0.787	0.784	0.937
	Nomogram	0.958	0.929-0.987	0.898	0.871	0.920	0.900	0.896
**Validation set**	Clinical	0.911	0.794-1.000	0.882	0.800	0.947	0.923	0.857
	Algorithm	0.968	0.915-1.000	0.941	0.933	0.947	0.933	0.947
	Nomogram	0.989	0.966-1.000	0.971	0.933	1.000	1.000	0.950

AUC, area under the receiver operating characteristic curve. ACC, accuracy; SEN, sensitivity; SPE, specificity; PPV, positive predictive value; NPV, negative predictive value.

### 3.2 Radiomics feature selection and radiomics models development

**Figure 3:**
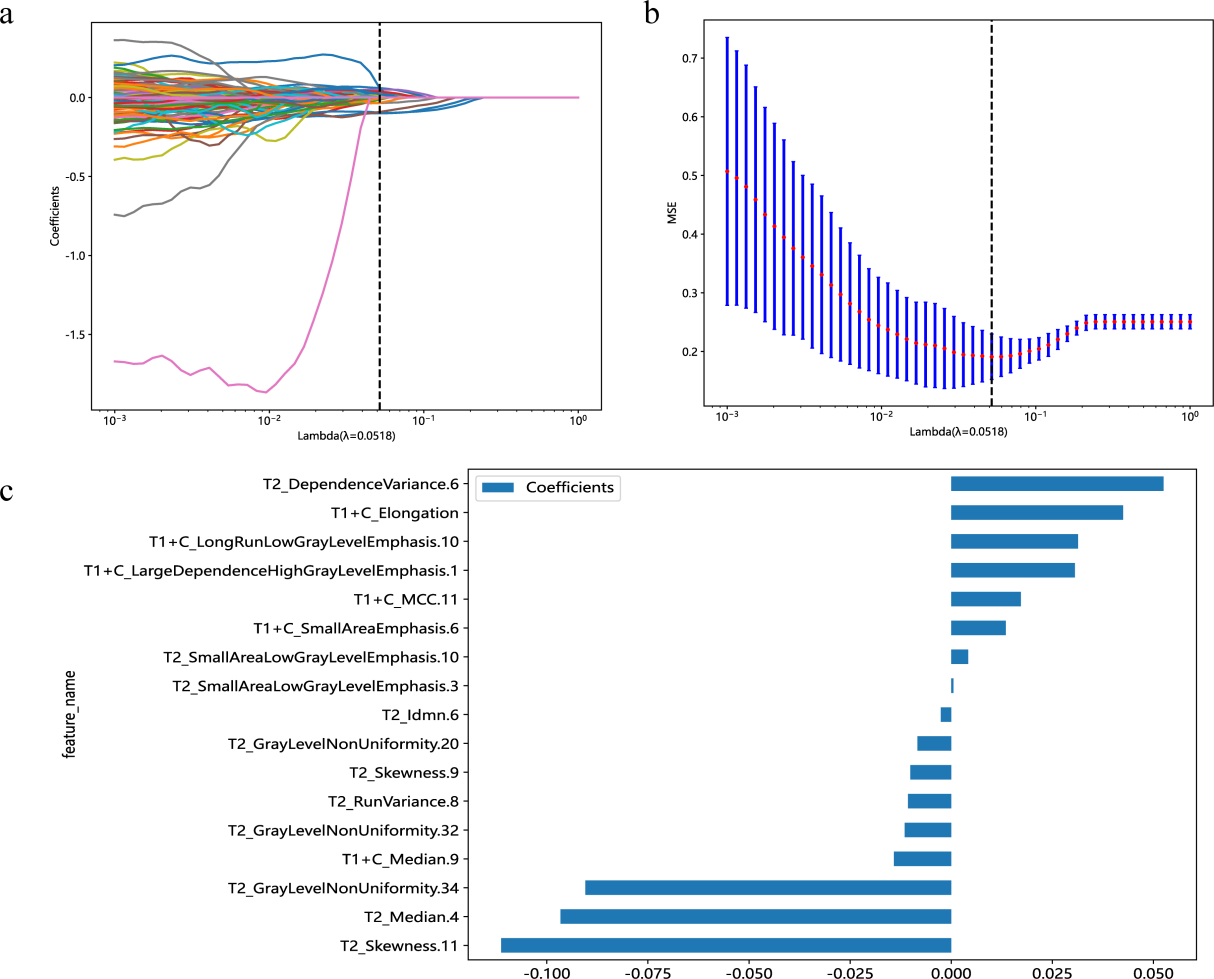
Predicated on the premise of an optimal λ value, delineated by a perpendicular line, a suite of 17 radiomic features was identified **(A)**. With modulation parameters (λ values), the various characteristics will affect the LASSO coefficients **(B)**. The selected 17 radiomics features and their nonzero coefficients **(C)**.

A total of 1,166 radiomics features, after confirming that both intraobserver and interobserver ICCs exceeded 0.750, were analyzed using the mRMR method, LASSO method and the Mann-Whitney U test. Ultimately, seventeen features revealed significant differences, encompassing 1 shape-based, 4 first-order statistics features, 2 GLCM features, 3 GLRLM features, 4 GLSZM features, and 3 GLDM features (**Figure 3**). Shape feature describes geometric parameters such as location and size of the lesion. The first-order feature describes the distribution of gray values of each voxel in the region of interest. Matrix-based features are second-order statistics that analyze the complexity of the structure inside and around the tumor, the variation of the layers, and the thickness of the texture. Regression analysis confirmed these features as independent predictors (P < 0.05). To establish the radiomics models, these seventeen characteristics were combined with SVM, KNN and LR. In regard to AUC and accuracy performance, the LR classifier achieved the maximum performance, yielding an AUC of 0.926 (95% CI, 0.885-0.967) in the training set and 0.968 (95% CI, 0.915-1.000) in the validation set. Correspondingly, accuracy rates were recorded at 0.854 and 0.941, respectively (**Table 4**). The texture feature model formula was as follows:

**Table 4:** Performance of the three machine-learning methods.

Classifier	Training set				Validation set			
	AUC (95% CI)	ACC	SEN	SPE	AUC (95% CI)	ACC	SEN	SPE
SVM	0.967 (0.943-0.991)	0.905	0.839	0.960	0.954 (0.886-1.000)	0.912	0.933	0.895
LR	0.926 (0.885-0.967)	0.854	0.935	0.787	0.968 (0.915-1.000)	0.941	0.933	0.947
KNN	0.912 (0.868-0.957)	0.847	0.855	0.840	0.935 (0.858-1.000)	0.882	0.800	0.947

AUC, area under the receiver operating characteristic curve; CI, confidence interval; ACC, accuracy; SEN, sensitivity; SPE, specificity.

Radscore = 0.438967728381277+0.000521*T2_SmallAreaLowGrayLevelEmphasis.3−0.096624*T2_Median.4+0.052464*T2_DependenceVariance.6−0.002580*T2_Idmn.6−0.008362*T2_GrayLevelNonUniformity.20−0.010714*T2_RunVariance.8−0.010133*T2_Skewness.9−0.011496*T2_GrayLevelNonUniformity.32+0.004185*T2_SmallAreaLowGrayLevelEmphasis.10−0.111251*T2_Skewness.11−0.090475*T2_GrayLevelNonUniformity.34+0.042483*T1+C_Elongation+0.030545*T1+C_LargeDependenceHighGrayLevelEmphasis.1+0.013493*T1+C_SmallAreaEmphasis.6−0.014165*T1+C_Median.9+0.031357*T1+C_LongRunLowGrayLevelEmphasis.10+0.017206*T1+C_MCC.11ccccc−4.130

### 3.3 Establishment and performance of nomogram

**Figure 4:**
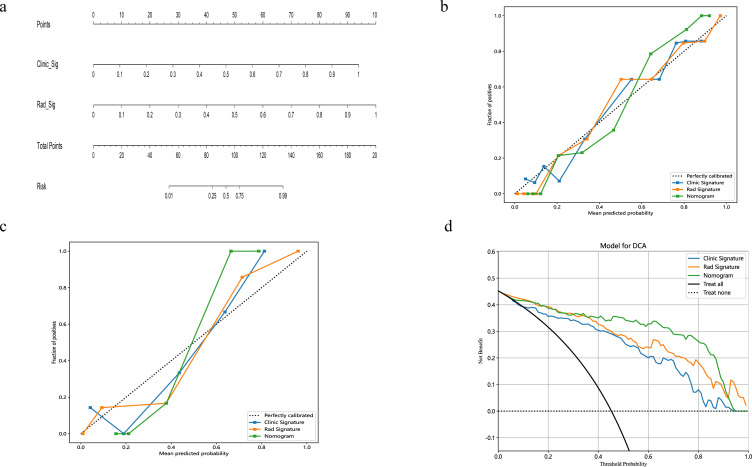
The nomogram integrates radiomic features with morphological characteristics **(A)**. Calibration curves for the radiomic nomograms in the training and validation cohorts are presented in **(B, C)**. A 45-degree line indicates perfect prediction; the closer the curve approximates this line, the greater the nomogram's predictive accuracy. Decision curve analysis (DCA) was employed for the radiomic nomogram **(D)**, with the net benefit plotted on the y-axis. In the nomogram, this is represented by a black line. The line labeled "All" assumes every patient has SFT, while the "None" line assumes no patients have SFT.

A radiomics nomogram, integrating age, width, dural tail sign, and rad-score, was established (**Figure 4**). Compared to the individual performance metrics of the radiomics algorithm (AUCs of 0.926 and 0.968 for the training and validation sets, respectively) and the clinical model (AUCs of 0.875 and 0.911), the nomogram exhibited superior predictive capabilities, with AUCs of 0.958 (95% CI, 0.929-0.987) and 0.989 (95% CI, 0.966-1.000) for the respective sets. Additionally, the nomogram demonstrated high accuracy (0.898), sensitivity (0.871), and specificity (0.920) in the training set, and similarly outstanding performance in the validation set (accuracy of 0.971, sensitivity of 0.933, specificity of 1.000) (**Table 3**, **Figure 2**). In the training group, the AUC of the nomogram was higher than that of both the clinical model and the radiomics model according to DeLong test. In the test group, there was no significant difference in AUC among the three groups (P > 0.05).

The calibration curves for the radiomics nomogram indicated excellent performance in both the training and validation sets (**Figure 4**). Furthermore, DCA of the nomogram revealed that within a threshold probability range of 0.15 to 0.85, and when comparing strategies of 'treat none' versus 'treat all', the nomogram consistently outperformed the clinical model (**Figure 4**).

## 4 Discussion

The preoperative distinction between SFT and AM is of paramount clinical significance. SFT, characterized by its invasive nature, has a propensity for recurrence and metastasis to extracranial organs post-surgical resection. Typically, the primary therapeutic strategy encompasses postoperative radiotherapy or chemotherapy, supplemented by regular clinical follow-ups to monitor patient prognosis (24). Conversely, AM exhibits a benign pathology, with low aggressive growth potential and recurrence rates, often resulting in favorable outcomes after gross total resection (25). SFT and AM exhibit similar MRI findings. Although previous studies have demonstrated that certain MRI features—such as tumor size, signal intensity, vascular flow voids, and the dural tail sign—can aid in differentiating between SFT and AM (26). However, traditional MRI features are susceptible to the influence of physician experience due to the lack of objectivity and quantitative analysis, so differentiating between these two entities using conventional MRI poses a considerable challenge. In our study, we selected the radiomics algorithm with superior predictive performance, integrating it with the clinical model to construct a radiomics nomogram specifically for distinguishing SFT from AM. The radiomics nomogram AUC was 0.989 in the validation set, outperforming the clinical model and the radiomics algorithm in predictive capability. Demonstrating satisfactory calibration and net benefits, the radiomics nomogram appears reliable for differentiating AM from SFT.

Image segmentation in machine learning for intracranial tumors, a crucial step, demands high reproducibility. Current methodologies range from manual to semiautomatic and fully automatic approaches (27, 28). Hu et al. (29) implemented a semiautomatic method based on a signal intensity threshold and edge-based algorithms via 3D Slicer, achieving efficient tumor segmentation. This approach automatically aligned the ROIs with multimodal MRI images, proving more efficient than manual sketching. However, despite its efficiency, automatic mapping encounters challenges, particularly with the variability in tumor morphology. In our study, manual segmentation was meticulously conducted using 3D Slicer to ensure precise tumor delineation.

The efficacy of morphological features in differentiating between AM and SFT remains contentious. Various studies advocate for their diagnostic relevance, noting characteristics typical of SFT such as large volume, irregular shape, uneven enhancement, vascular flow voids, dural stenosis, necrosis, and bone deterioration (19, 30–33). Contrarily, our research suggests a divergence from these findings, potentially due to the subjective nature of clinical observations. Our multivariate logistic regression analyses identified age, width, and dural tail sign as independent predictors in distinguishing SFT from AM. In addition, the AUC of the clinical model in our validation set was inferior to that of the radiomics algorithms and nomograms, underscoring the dependency of the clinical model's performance on radiologist expertise and emphasizing the superior discriminative performance of machine learning algorithms (33).

Radiomics, a constantly evolving new subject in medical imaging, refers to the quantitative extraction of radiological features from two-dimensional or three-dimensional medical images. Its integration with clinical data has proven beneficial in monitoring tumor progression and aiding personalized treatment strategies (34). Machine learning plays a crucial role in analyzing brain tumor MRI imaging data, facilitating deeper exploration of patterns that can aid in diagnosis. At present, domestic and foreign scholars have applied machine learning based on radiomics methods to study SFT and AM. Fan et al. (35) utilized SVM to develop a model for distinguishing SFT from AM using CE-T1WI, T2WI, and a combined CE-T1WI and T2WI sequence. The combined CE-T1WI and T2WI model demonstrated the highest predictive performance, achieving an AUC of 0.90. Kong et al. (36) assessed various algorithms' effectiveness in differentiating SFT from AM using multiparameter MRI sequences. Their results indicated that the performance improvements offered by the different algorithms were limited. However, most studies typically utilize only one machine learning algorithm or a single traditional MRI sequence to construct their models, often using data from a single center. Validating the generalizability of these models necessitates the inclusion of multicenter data. Therefore, in line with previous research, we integrated data from multiple centers and employed LR, SVM, and KNN algorithms in conjunction with conventional MRI sequences to establish radiomics models, and the models ultimately showed robust predictive capabilities. Furthermore, our integrated model demonstrated superior diagnostic accuracy, outperforming the individual LR, SVM, and KNN models, as well as the clinical model. This underscores that while tumor radiomics models possess enhanced predictive abilities compared to the clinical model alone, the integration of clinical data is indispensable. The synergy of these elements effectively differentiates between SFT and AM. A radiomics nomogram, established by Wei et al. to differentiate SFT from AM, showcased remarkable diagnostic capability, with AUCs of 0.985 and 0.917 in the training and validation sets, respectively (37).

In our study, 1,166 radiomics features, predominantly texture features representing second-order attributes that reflect voxel/pixel relationships, were extracted. Texture features illustrate not only pixel intensity distribution, but also how quantized pixels position each other (38). The feature selection process in machine learning, crucial for the reliability of radiomics algorithms, involved the use of mRMR and LASSO. LASSO is particularly effective in datasets with numerous features but limited samples without noticeably increasing the bias, while mRMR facilitates the selection of features with minimal redundancy and maximum relevance (39, 40). Subsequently, seventeen features were selected to develop radiomics models using SVM, LR, and KNN classifiers. SVM is adept at analyzing high-dimensional, small-scale data. KNN excels in processing nonlinear data, identifying multiple predictive biomarkers in clinical datasets. LR, as a classification method, investigates the relationship between specific outcome probabilities and features (41, 42). Our results indicated that LR yielded the highest accuracy and AUC, prompting their adoption for the optimal radiomics model. Integrating this model with the clinical model to create the radiomics nomogram resulted in excellent calibration and accurate differentiation between SFT and AM in the validation set, affirming its efficacy and reliability in diagnosing these conditions.

Multicenter studies, essential for obtaining extensive sample sizes and enhancing classifier generalization, encounter challenges like data heterogeneity and variability arising from different scanners and methodologies. To mitigate these issues, we implemented preprocessing steps such as resampling, denoising, and wavelet transformation. In the validation set, clinical model, radiomics algorithm, and the nomogram demonstrated AUCs of 0.911, 0.968, and 0.989, respectively, suggesting robust generalizability (43).

This investigation had certain restrictions. Firstly, its retrospective nature and relatively small size might introduce selection bias. Considering the retrospective design, scanner and protocol heterogeneity were addressed through resampling to stabilize model performance. Additionally, patient distributions varied between training and validation sets. Lastly, our analysis was confined to images from the CE-T1WI and T2WI. Future studies aim to include a broader range of imaging sequences to further enhance the predictive accuracy and generalizability of the model.

## 5 Conclusions

In summary, compared to conventional MRI, the MRI-based radiomics nomogram demonstrates greater efficacy in differentiating SFT and AM, offering significant information for the subsequent treatment and detection. Furthermore, the study of a larger prospective dataset is needed to certify the real value of nomogram.

## Data Availability

The raw data supporting the conclusions of this article will be made available by the authors, without undue reservation.
